# Muscle Strength and Left Ventricular Systolic and Diastolic Dysfunction in Chronic Kidney Disease Men: A Pilot Study

**DOI:** 10.3390/jcm15041338

**Published:** 2026-02-08

**Authors:** Katarzyna Romejko, Katarzyna Szamotulska, Stanisław Niemczyk

**Affiliations:** 1 Department of Internal Diseases, Nephrology and Dialysis, Military Institute of Medicine—National Research Institute, 128 Szaserów Street, 04-141 Warsaw, Poland; sniemczyk@wim.mil.pl; 2 Department of Epidemiology and Biostatistics, Institute of Mother and Child, 17a Kasprzaka Street, 01-211 Warsaw, Poland; katarzyna.szamotulska@imid.med.pl

**Keywords:** sarcopenia, hand grip strength, five-times sit-to-stand test, chronic kidney disease, left ventricular diastolic dysfunction

## Abstract

**Background:** Sarcopenia is defined by decreased muscle strength along with low muscle quantity or quality. The assessment of muscle strength may be performed by grip strength test or chair stand test (CST) and both of these tests are treated as equivalent tools for assessing muscle strength. Heart failure with preserved ejection fraction (HFpEF) contributes to the progression of sarcopenia, and it is left ventricular diastolic dysfunction (LVDd) which primarily leads to the development of HFpEF. The aim of this study was to examine the relationship of muscle strength with echocardiographic parameters of LVDd in patients with CKD and eGFR ≤ 29 mL/min/1.73 m^2^ not treated with dialysis. **Methods:** The study samples consisted of 46 men with CKD stages G4–G5 not treated with dialysis: 23 participants with HGS < 27 kg and 23 individuals with HGS ≥ 27 kg. The assessment of muscle strength was provided by the hand grip strength (HGS) test and the five-times sit-to-stand test (FTSST). Transthoracic echocardiography was performed with the use of a convex probe in conjunction with a Logiq P6 ultrasound system. **Results:** In G4–G5 CKD patients, upper limb muscle strength did not correspond to lower limb muscle strength. Participants with prolonged FTSST had a lower mean value of septal e’ and higher mean E/e’ compared to individuals with correct both HGS and FTSST. Participants with correct HGS and prolonged FTSST had the lowest mean left ventricular ejection fraction (LVEF), as well as the lowest mean tricuspid annular plane systolic excursion (TAPSE). **Conclusions:** In G4–G5 CKD patients not treated with dialysis, HGS and FTSST are not equivalent and should not be used interchangeably. In this population, decreased muscle strength is associated with LVDd and FTSST is more sensitive than HGS in the prediction of LVDd. Low muscle strength is also associated with systolic function of the left and right ventricle in G4–G5 CKD patients not treated with dialysis.

## 1. Introduction

Sarcopenia is defined as a loss of proper muscle architecture, decreased muscle mass, and impaired muscle function and strength. The diagnostic criteria of sarcopenia in the general population are based on the updated guidelines of the European Working Group on Sarcopenia in Older People (EWGSOP2) 2018 [1]. As decreased muscle strength is the most reliable tool for assessing muscle function, sarcopenia is probable if decreased muscle strength is diagnosed. Sarcopenia is confirmed by the presence of low muscle quantity or quality and is severe when accompanied by low physical performance. Sarcopenia is a common finding in chronic kidney disease (CKD); the prevalence of sarcopenia in this group of patients exceeds 25% and is highest in patients treated with dialysis [2]. Due to the lack of specific diagnostic criteria for patients with kidney function decrease, the diagnosis of sarcopenia in this group is based on EWGSOP2. However, the mechanisms of decreased muscle strength, muscle function and muscle mass in the elderly population and in CKD are not entirely the same. The causes of sarcopenia development in CKD are multivarious and include increased inflammatory processes, insulin resistance, hyperparathyroidism, resistance to growth hormone (GH) and impaired response to growth hormone—insulin-like growth factor-1 (GH/IGF-1)—the derangements of adipocytokine profile, hypogonadism with decreased serum testosterone concentrations, and also dietary restrictions and apetite disorders [3].

Sarcopenia leads to decreased physical activity, high susceptibility to injuries, increased number of hospitalizations and elevated mortality [4]. The changes in muscle function and muscle structure are also considered as crucial determinants of heart failure pathophysiology and progression [5]. On the other hand, the multivarious pathological states that are present in heart failure such as hormonal changes, malnutrition, oxidative stress, increased inflammatory processes, endothelial dysfunction and reduced muscle blood flow significantly contribute to the development of sarcopenia [6]. Thus, chronic heart failure and sarcopenia are linked by a reciprocal relationship [7].

Heart failure (HF), including heart failure with preserved ejection fraction (HFpEF), is one of the main cardiovascular complications in CKD and it is left ventricular diastolic dysfunction (LVDd) that primarily leads to the development of HFpEF. The causes of LVDd in CKD are multivarious, including arterial hypertension, concomitant diabetes, erythropoietin and vitamin D deficiency, proteinuria, increased uremic toxins, elevated serum concentrations of fibroblast growth factor 23 (FGF23) and advanced glycation end-products (AGEs). Increased subclinical inflammation in CKD causes endothelial dysfunction, enhances endothelial inflammatory processes and inflammatory cells infiltration and thus consequently promotes myocardial fibrosis which leads to LVDd [8]. Additionally, recent studies indicate that sarcopenia in elderly groups of patients is also a risk factor of LVDd [9]. However, the number of studies which examine the association of sarcopenia or its components such as muscle strength and muscle mass with LVDd parameters in the CKD population is limited.

According to EWGSOP2 guidelines, the estimation of muscle strength may be performed by the grip strength test or chair stand test (CST). The EWGSOP2 recommendations underline that grip strength correlates with strength in other body compartments and may be a surrogate for other measurements of upper and lower limb strength [1]. Thus, according to EWGSOP2, those two tests may be used interchangeably. However, in populations different from CKD patients there are studies the results of which confirmed that these two tests are not equivalent [10,11].

The aim of this pilot study was to examine the relationships of muscle strength with the echocardiographic parameters of LVDd in patients with CKD and eGFR ≤ 29 mL/min/1.73 m^2^ not treated with dialysis and to formulate detailed hypotheses regarding these associations.

## 2. Materials and Methods

### 2.1. Patients

The study samples consisted of 46 men with CKD stages G4–G5 not treated with dialysis (23 participants with HGS < 27 kg and 23 individuals with HGS ≥ 27 kg according to EWGSOP2 criteria). Patients were recruited to the study from the Nephrological Outpatient Clinic of the Military Institute of Medicine—National Research Institute in Warsaw, Poland. The inclusion criteria were age between 40 and 80 years and CKD stages G4–G5. Individuals aged below 40 or over 80 years, those with eGFR ≥ 30 mL/min/1.73 m^2^, patients treated with dialysis or requiring initiation of dialysis within three months after recruitment, men with clinical signs of infections, with the presence of metal parts in the body, and patients after physical exertion or alcohol consumption on the day prior to the examination were excluded from the study. Each participant signed an informed consent before beginning of the study. The study protocol was accepted by the Bioethics Committee in Military Institute of Medicine—National Research Institute in Warsaw, Poland (Institutional Review Board acceptance number KB/6/23, obtained 20 December 2023), which means that the study was performed in accordance with the ethical standards established in the 1964 Declaration of Helsinki and its later amendments.

### 2.2. The Assessment of Sarcopenia Risk Factors

Nutritional and non-nutritional risk factors of sarcopenia were assessed with the use of Polish validated version of the Mini Sarcopenia Risk Assessment Questionnaire (PL-MSRA-7). The total score of ≤30 in PL-MSRA-7 indicates the risk of sarcopenia [12].

### 2.3. The Assessment of Muscle Strength

The assessment of upper limb muscle strength was provided by hand grip strength (HGS) test with the use of calibrated dynamometer. HGS was evaluated in a seated position, with the arm adducted, the elbow flexed at 90°, and the forearm in a neutral position. The wrist was maintained in a neutral alignment, defined as 0–30° of dorsiflexion and 0–15° of ulnar deviation. Three trials were performed for each hand and the highest value obtained from the dominant hand was recorded for analysis. The cut-off point for decreased HGS for men was <27 kg [1].

Lower limb muscle strength was measured with the use of the five-times sit-to-stand test (FTSST). Participants were seated on a standard chair (seat height approximately 46–48 cm), with their arms crossed over the chest. On the examiner’s command, they were instructed to stand up fully and sit down five times as quickly as possible. The outcome measure was the time, measured in seconds, required to complete five repetitions. The cut-off point for decreased lower limb muscle strength evaluated by FTSST was >15 s [1].

### 2.4. The Assessment of Muscle Mass and Body Composition Parameters

Body composition parameters including lean tissue mass (LTM), lean tissue index (LTI), fat mass (Fat), fat tissue index (FTI) and overhydration (OH) were measured by bioimpedance spectroscopy (BIS) using Body Composition Monitor (BCM) (Fresenius Medical Care, Bad Homburg, Germany). Patients remained in a supine position with electrodes placed in a tetrapolar configuration (on one hand and one foot). LTI was calculated as lean tissue mass in kilograms and FTI was calculated as fat mass in kilograms, both divided by height in square meters. Muscle mass was calculated from BCM using the Lin’s algorithm, which derived a formula for appendicular skeletal muscle mass (ASM) estimation based on parameters obtained from BIS [13].

### 2.5. The Assessment of eGFR

The estimated glomerular filtration rate (eGFR) was calculated using the 2021 Chronic Kidney Disease Epidemiology Collaboration (CKD-EPI) creatinine equation:

*eGFRcr* = 142 × *min* (*Scr*/*κ*, *1*) *α* × *max* (*Scr*/*κ*, *1*) − 1200 × 0.9938 *Age*

Scr = serum creatinine in mg/dL; κ = 0.9; α = −0.302; min (Scr/κ, 1); and max (Scr/κ, 1) represent the smaller and larger of Scr/κ or 1.0, respectively; Age = age in years.

### 2.6. Laboratory Parameters

Blood samples were collected after a 12 h overnight fast and were transported to the local Department of Laboratory Diagnostics. Serum total cholesterol, creatinine, albumin, and glucose were determined using enzymatic colorimetric (CHOD-PAP), enzymatic, colorimetric with bromocresol green (BCG), and enzymatic hexokinase methods, respectively, on the Cobas c 503 PRO analyzer (Roche Diagnostics, Mannheim, Germany). Serum testosterone and insulin concentrations were measured by electrochemiluminescence immunoassay (ECLIA), on the Cobas e 801 PRO analyzer (Roche Diagnostics, Mannheim, Germany). Haemoglobin concentration was determined by the sodium lauryl sulfate (SLS) method using the SYSMEX XN-1000 analyzer (Sysmex Corporation, Kobe, Japan).

### 2.7. The Assessment of Echocardiography Examination

A transthoracic echocardiography was performed with the use of convex probe in conjunction with a Logiq P6 ultrasound system (GE Healthcare, Seoul, Korea). Echocardiographic measurements including two-dimensional and Doppler studies were obtained in accordance with the American Society for Echocardiography guidelines for obtaining images, chamber dimensions and assessment of transvalvular flow [14]. Left ventricular ejection fraction (LVEF) was assessed using the modified biplane Simpson’s method. Left ventricular end-diastolic volume (LVEDV) and left ventricular end-systolic volume (LVESV) were obtained from apical four-chamber and two-chamber views. LVEF was calculated according to the following formula: LVEF = (LVEDV − LVESV)/LVEDV × 100%. LVDd was evaluated by the following parameters: early diastolic velocity at the lateral mitral annulus (lateral e’), early diastolic velocity at the septal mitral annulus (septal e’), average e’ = (septal e’ + lateral e’)/2, average septal-lateral E/e’ ratio (E/e’), left atrial volume index (LAVI), left ventricular mass index (LVMI), relative wall thickness (RWT), interventricular septum (IVS), posterior wall diameter (PWD), the maximal velocity of early diastolic transmitral inflow (E wave), the ratio of the maximal velocity of early diastolic transmitral inflow and the maximal velocity through mitral valve in the later atrial contraction (E/A). The systolic function of the right ventricle was assessed based on tricuspid annular plane systolic excursion (TAPSE) and pulsed wave–Doppler tissue imaging (PW–DTI)-derived tricuspid lateral annular peak systolic velocity (S’ wave).

Two patients from the study group died before performing echocardiography examination and the physical condition of one participant from the study group deteriorated to such an extent that he was unable to attend echocardiography. Hence, the total number of patients with performed echocardiography examination was 20 in the study group and 23 in the control group. Additionally, one patient from the control group had permanent atrial fibrillation, which prevented the estimation of all assessed parameters of the left ventricular diastolic function.

### 2.8. Statistical Analysis

Descriptive statistics (mean, standard deviation, quartiles, proportion) were applied in the groups. Continuous variables were compared between two groups using the exact Mann–Whitney test. For subgroup analysis, the exact Kruskal–Wallis test with pairwise comparisons adjusted by the Bonferroni correction for multiple tests was applied. Categorical variables were compared using chi-square or Fisher test. A *p*-value < 0.05 was considered to be statistically significant. Statistical analyses were performed using IBM SPSS v. 29.0 (Armonk, NY, USA).

## 3. Results

The study included 23 men with low HGS (<27 kg), the study group, and the control group, 23 individuals with correct HGS (≥27 kg). Both samples were taken from the population of patients with severely decreased eGFR stages G4–G5 of CKD not treated with dialysis, of similar age [15]. The mean age in the study group was 65 years and in the control group it was 63 years.

Patients with decreased and correct HGS did not differ significantly in terms of the average concentration of considered biochemical parameters such as serum creatinine, eGFR, serum glucose, insulin, homeostasis model assessment of insulin resistance (HOMA-IR), haemoglobin, serum albumin, total cholesterol and serum testosterone (Table 1). However, in the group of patients with HGS < 27 kg, 52.2% of participants had fasting serum glucose above 100 mg/dL compared to 39.1% of men in the control group. Mean plasma insulin and HOMA-IR were higher in the study group and HOMA-IR above 2.5 was observed in 47.8% of participants from the study group versus 43.5% of men from the control group. A total of 43.5% of patients with HGS < 27 kg and 34.8% of individuals with HGS ≥ 27 kg had previously diagnosed diabetes. Mean plasma albumin was higher in the control group, and the difference was at the border of significance (*p* = 0.055). Additionally, 21.7% of patients with HGS ≥ 27 kg had serum total cholesterol concentrations above 190 mg/dL compared to 13.0% of participants with HGS < 27 kg. We also found that in the group of patients with lower HGS, 34.8% of men had serum testosterone concentrations below 2.8 ng/mL, compared to individuals with correct HGS, where decreased plasma testosterone levels were observed in 21.7% of patients.

Although average values of anthropometric variables such as weight, height and body mass index (BMI) were similar in both groups, more patients with correct HGS were obese (34.8%) compared to participants in the study group (26.1%) and the opposite trend was observed for BMI below 24.9 kg/m^2^ (30.4% of patients from the study group and 26.1% of participants from the control group). The mean values of body composition parameters such as LTM, LTI, Fat, FTI, ASM and OH were also similar in the two groups (Table 1); however, increased fluid overload (OH over 1 L) was found in 69.6% of participants in the study group and in 52.2% of individuals in the control group.

Patients with decreased HGS had higher numbers of hospitalizations in the year preceding the study compared to participants with the correct HGS (*p* = 0.036). Also, in the group with HGS < 27 kg, 56% of individuals were unable to walk more than 1000 m, which was significantly higher compared to 13% in the group with HGS ≥ 27 kg (*p* = 0.002) (Table 2).

Arterial hypertenstion was diagnosed in 65.2% of patients from the group with HGS < 27 kg and in 60.9% of participants with HGS ≥ 27 kg (*p* = 1.000). Heart failure was reported in 60.9% of individuals with decreased HGS and in 30.4% of men with correct HGS (*p* = 0.075). Coronary artery disease and history of myocardial infarction was noted in 60.9% and 34.8% of patients from the study group and in 52.2% and 17.4% of participants from the control group, respectively (*p* = 0.767 and *p* = 0.314).

We did not observe statistically significant differences between the two groups of participants according to the echocardiographic parameters. Nonetheless, we found that the septal e’ was lower in patients with HGS < 27 kg, as was lateral e’. In the group of participants with HGS < 27 kg, more patients had septal e’ lower than 7 than in the group of men with HGS ≥ 27 kg. The mean value of E/e’ was slightly higher in the group of patients with HGS < 27 kg. We also observed that more participants with HGS < 27 kg had E/e’ ≥ 9 than individuals with HGS ≥ 27 kg. Additionally, the mean value of RWT was higher in patients with HGS < 27 kg compared to participants with HGS ≥ 27 kg. We found that more patients with HGS < 27 kg had RWT > 0.42 than participants with HGS ≥ 27 kg (Table 3).

**Figure 1:**
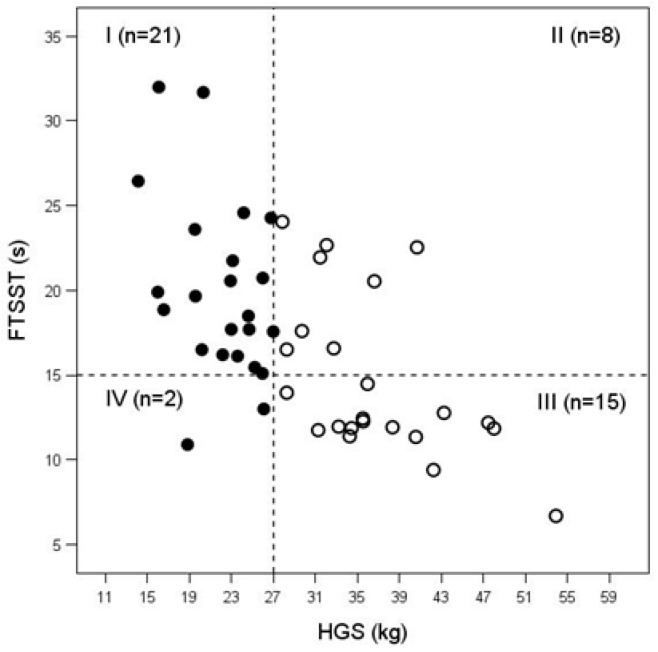
HGS and FTSST in patients with CKD stages G4–G5 in subgroups I, II, III, IV (● study group, ○ control group).

In our study, we noticed that in CKD patients stages G4–G5 of upper limb muscle strength assessed with HGS did not strictly correspond to lower limb muscle strength evaluated by FTSST, which means that these two measurements of muscle strength are not equivalent in CKD patients (Figure 1). As in our study some patients with correct HGS had prolonged FTSST, we performed subgroup analysis classifying each of the studied samples into two subgroups according to the results of the FTSST. We obtained four sets of observations: individuals with decreased HGS (<27 kg) and with prolonged FTSST (>15 s)—group I (n = 21), men with correct HGS (≥27 kg) but with increased FTSST (>15 s)—group II (n = 8), participants with correct strength of upper and lower limbs (HGS ≥ 27 kg and FTSST ≤ 15 s)—group III (n = 15), and patients with low HGS (<27 kg) and correct FTSST (≤15 s)—group IV (n = 2). Group IV was excluded from the subgroup analysis due to the extremely low number of patients.

By analysing the subgroups we observed that patients with the correct HGS and prolonged FTSST (group II) had the lowest mean LVEF (*p* = 0.021). They had also the highest proportion of LVEF below 50%. Similar results were observed for the systolic function of the right ventricle. We found that participants with the correct HGS and prolonged FTSST (group II) had the lowest mean TAPSE (*p* = 0.017) and the highest proportion of TAPSE lower than 18 mm (Table 4). Overall, LVEF below 50% and/or TAPSE below 18 mm were the most common in group II (*p* = 0.055 in the Fisher test).

The parameters of LVDd were also different in patients with correct versus prolonged FTSST. Participants with correct both HGS and FTSST (group III) had a higher mean value of septal e’ compared to individuals with prolonged FTSST (independently of HGS) (*p* = 0.016). They had also lower proportions of septal e’ below 7 cm/s in comparison to groups I and II. Also, the mean average e’ was found to be higher in patients with the correct both HGS and FTSST (group III) compared to participants with prolonged FTSST, both with proper and decreased HGS, but the results were at the border of significance (*p* = 0.057). Additionally, mean E/e’ was lower in the group of patients with proper upper and lower limb muscle strength (group III) compared to participants with prolonged FTSST (independently of HGS) (*p* = 0.030) (Table 4). A much smaller proportion of participants from group III had E/e’ ≥ 9 than patients with prolonged FTSST (independently of HGS). The highest LAVI and the highest proportion of LAVI ≥ 29 mL/m^2^ was observed in group II, whereas in the remaining groups the proportion of LAVI ≥ 29 mL/m^2^ was approximately twice lower. Mean lateral e’ was also the lowest in group II; however, in groups I and III it was also below 10 cm/s. The proportion of lateral e’ below 10 cm/s was highest in group II. Similarly, mean LVMI and the proportion of LVMI above 115 g/m^2^ were highest in group II. Regarding RWT, in group I and group II the proportions of RWT above 0.42 were higher compared to participants with correct both HGS and FTSST. Overall, the mean number of LVDd parameters was the highest in group II in which it equalled to 3.71 ± 1.70, and in group I it was 3.28 ± 1.27, whereas in the group with correct both HGS and FTSSS it equalled 2.20 ± 1.74 (*p* = 0.082 in the exact Kruskal–Wallis test).

Arterial hypertenstion was diagnosed in 61.9% of patients from group I, 62.5% of participants from group II and 60.0% of individuals from group III (*p* = 1.000). Heart failure was reported in 66.7%, 50.0% and 20.0% of patients from groups I, II, and III, respectively (*p* = 0.018). Coronary artery disease was diagnosed in 61.9% of participants with HGS < 27 kg and FTSST > 15 s, 75.0% of men with HGS ≥ 27 kg and FTSST > 15 s, and 40.0% of individuals with HGS ≥ 27 kg and FTSST ≤ 15 s (*p* = 0.262). The history of myocardial infarction was noted in 33.3%, 25.0% and 13.3% of patients from groups I, II, and III, respectively (*p* = 0.419).

We did not observe statistically significant differences between the three groups in terms of the laboratory parameters. However, average serum glucose concentrations were highest in patients with the correct HGS and prolonged FTSST (group II). A total of 33.3% of patients with HGS < 27 kg and FTSST > 15 s (group I) had serum glucose concentrations ≥ 126 mg/dL, in participants with HGS ≥ 27 kg and FTSST > 15 s (group II) 50% of individuals had serum glucose levels ≥ 126 mg/dL, and only 6.7% of patients with correct both HGS and FTSST had serum glucose concentrations ≥ 126 mg/dL (*p* = 0.045). Similar results were found for mean insulin concentrations and mean HOMA-IR, the highest values of which were observed also in group II. Additionally, 42.9% of participants with HGS < 27 kg and FTSST > 15 s (group I) had HOMA-IR > 2.5, in the group of patients with HGS ≥ 27 kg and FTSST > 15 s (group II) 75% of individuals had HOMA-IR > 2.5, and only 26.7% of patients with HGS ≥ 27 kg and FTSST < 15 s had HOMA-IR > 2.5. Previously diagnosed diabetes was present in 42.9% of patients in group I, 75.0% in group II and 13.3% in group III. Patients with HGS ≥ 27 kg, both with prolonged and proper FTSST, had higher mean serum total cholesterol and albumin concentrations than men with decreased HGS. Mean plasma testosterone levels were also higher in participants with HGS ≥ 27 kg (Table 5).

We did not observe statistically significant differences in anthropometric parameters such as ASM and BMI. However, patients with HGS ≥ 27 kg and FTSST > 15 s (group II) had the highest average weight, were the tallest and had the highest amount of fat (Fat and FTI). According to BMI, 62.5% of them had BMI ≥ 30 kg/m^2^, compared to 23.8% of participants in group I and 20% of men in group III. We also observed that LTM and LTI were higher in patients with correct HGS and FTSST (group III) compared to individuals with prolonged FTSST, both with HGS < 27 kg and HGS ≥ 27 kg (Table 5).

We observed that the highest proportion of participants with impaired both HGS and FTSST (group I) were unable to walk more than 1000 m compared to individuals with the correct HGS (*p* = 0.008). The number of hospitalizations during the year preceding the survey was also the highest in group I. However, we found no statistically significant differences in the frequency and the types of meals consumed between the three groups of patients. The groups also did not differ significantly regarding weight loss in the preceding year (Table 6).

## 4. Discussion

In our study, we did not demonstrate the statistically significant differences between CKD patients in stages G4–G5 not treated with dialysis with HGS < 27 kg and individuals with HGS ≥ 27 kg regarding echocardiographic, clinical and anthropometric characteristics. However, we observed higher proportions of LVDd parameters such as septal e’, E/e’ and RWT in patients with HGS < 27 kg in comparison to participants with HGS ≥ 27 kg. Both septal e’ lower than 7 cm/s and RWT above 0.42 are the major diagnostic criteria for LVDd. A value of E/e’ between 9 and 14 is a minor criterion for LVDd [16].

In the general elderly population, muscle strength can be assessed with the use of HGS or CST which may be performed interchangeably [1]. However, our subgroup analysis showed that in CKD patients both tests which evaluate upper and lower limb muscle strength such as HGS and FTSST were not replaceable. There are studies which support our results. The study of Belfield revealed that in a population of diabetic patients, the assessment of muscle strength with CST was associated with a higher prevalence of sarcopenia compared to HGS and concluded that those two tests should not be used interchangeably in this population [10]. The report of Johansson also found that HGS and CST differ and using CST more than doubled the prevalence of probable sarcopenia [11]. Verstraeten confirmed that HGS and CST are not equivalent as diagnostic measurements for probable sarcopenia in geriatric populations. However, they observed that decreased HGS, but not prolonged CST, was associated with higher hospitalisation and mortality rates [17]. Similarly to Verstraeten, Li observed that in the elderly Chinese population the prevalence of possible, confirmed and severe sarcopenia was significantly higher when using HGS compared to CST [18]. The results of recent studies in different groups of patients confirm that HGS and CST are not equivalent and should not be used interchangeably, which we also observed in G4–G5 CKD patients not treated with dialysis.

Our subgroup analysis showed that the mean value of septal e’ was lower in patients with prolonged FTSST (independently of HGS) in comparison with individuals with correct both HGS and FTSST (*p* = 0.016). Similarly, E/e’ was higher in patients with prolonged FTSST (independently of HGS) compared to participants with correct both HGS and FTSST (*p* = 0.030). Additionally, we observed that the proportions of LVDd parameters such as septal e’ below 7 cm/s and E/e’ ≥ 9 were higher in patients with prolonged FTSST (independently of HGS) than in individuals with correct both HGS and FTSST. We may therefore conclude that it is FTSST whose prolonged value shows higher sensitivity in predicting LVDd in patients with CKD stages G4–G5 not treated with dialysis.

The studies evaluating muscle strength and LVDd are sparse. The study of Beyer revealed that higher HGS was associated with lower left ventricular mass and lower left ventricular mass to volume ratio assessed by cardiovascular magnetic resonance, which may reflect the relationship between muscle strength and cardiac remodelling [19]. Since left ventricular muscle mass is currently also a diagnostic criterion for LVDd, we may assume that the study of Beyer was the first report which found the association between skeletal muscle strength and LVDd [16,19]. The study of Markus found an inverse relationship between HGS and left ventricular diastolic stiffness as well as between HGS and the serum concentrations of N-terminal pro-B-type natriuretic peptide (NT-proBNP) in patients aged 20–93 years [20]. The increased plasma NT-proBNP is currently also a major or minor criterion for LVDd, depending on the value of its serum concentrations [16]. Bekfani also observed that HGS was the lowest in the group of patients with E/e’ ≥ 15 compared to those with E/e’ ≤ 8 and individuals with E/e’ between 9 and 14 [21]. Additionally, there are studies which found that sarcopenia diagnosed as both decreased muscle strength and muscle mass is associated with LVDd, also in CKD patients [22,23]. Nevertheless, our study is the first which evaluates the association of muscle strength and numerous parameters of LVDd and also compares two methods of muscle strength assessment such as HGS and FTSST and their relationship with LVDd parameters in CKD patients not treated with dialysis.

As patients with prolonged FTSST (independently of HGS) had more parameters of LVDd relative to individuals with proper both HGS and FTSST, we compared those groups based on anthropometric, body composition and clinical variables. Although we did not find statistically significant differences between the groups, we observed that patients from group II were the heaviest and tallest, and had the highest BMI and ASM, the amount of fat and the value of FTI. In our previous study, we found that HGS significantly and positively correlated with body mass and height in CKD participants with eGFR below 45 mL/min/1.73 m^2^ not treated with dialysis [24]. Also, the study of Pua found that HGS was positively associated with height, weight and BMI in adults over 50 years of age [25]. Xu observed that in younger adults HGS correlated positively with weight; however, Byambaa found that it was height which was significantly associated with HGS [26,27]. Some studies involving patients with CKD confirmed that HGS was associated with anthropometric parameters as well. Hasheminejad observed a significant positive association between HGS and height, and also between HGS and weight in CKD populations [28]. Hernández Corona also found that in CKD patients HGS was related with both weight and height [29]. The above results from other studies suggest that correct HGS in group II might be associated with higher values of anthropometric variables such as weight and height. In contrast to HGS, the decreased lower limb muscle strength measured by FTSST was observed in obese patients [30]. The study of Lázaro-Martinez also found that prolonged CST was positively associated with the percentage of fat mass, which was confirmed in the study of de Paula Chaves Freitas [31,32]. Michou reported that in CKD patients decreased lower limb muscle strength was accompanied by higher percentages of fat mass [33]. These observations suggest that prolonged FTSST in group II may be associated with higher BMI and higher proportions of fat. According to the clinical parameters, average serum glucose and insulin and the value of HOMA-IR were also the highest in group II, and diabetes was previously diagnosed in the highest proportion of patients from group II (*p* = 0.011), which suggests that it was diabetic status which might have an influence on prolonged FTSST in group II. Indeed, patients with diabetes are observed to have decreased lower limb muscle strength as well as worse HGS [34,35,36]. Considering all metabolic disorders in group II like the highest BMI, the percentage of fat, FTI, the highest average serum glucose and insulin, the highest value of HOMA-IR and the highest proportion of patients with diabetes, we may assume that these metabolic disorders may have detrimental effects on FTSST. It is also worth mentioning that group II had on average the highest number of LVDd parameters such as LAVI, septal e’, lateral e’, E/e’, LVMI and RWT which suggests the association of LVDd with metabolic disturbances, including diabetes. LVDd may also impair FTSST. According to EWGSOP2 guidelines and taking into account HGS (not FTSST) in this group, these patients would be treated as healthy individuals, without even probable sarcopenia. However, they are patients with metabolic disturbances and cardiovascular complications, which, taking into account FTSST (not HGS), may be identified as probable.

Based on the systolic function of the left ventricle, we found that patients with correct HGS and prolonged FTSST (group II) had the lowest mean LVEF (*p* = 0.021) and the highest proportion of LVDd below 50%. To date, only a limited number of studies have directly investigated the relationship between muscle strength and LVEF. In the study of Zhu, the positive association between HGS and LVEF in male populations was found [37]. Bayer reported the positive relationship between HGS and left ventricular stroke volume [19]. Triangto revealed that CST may be used to assess cardiorespiratory fitness in patients with systolic dysfunction of the left ventricle [38]. We did not find similar studies which evaluated the direct relationship between muscle strength and LVEF in CKD populations. Additionally, patients with correct HGS and prolonged FTSST (group II) had the lowest mean TAPSE (*p* = 0.017) and the highest proportion of TAPSE ≤ 18 mm compared to the remaining groups. Although there are no studies directly linking TAPSE with HGS or CST, there is evidence that the improvement of muscle strength may influence right ventricular function [39].

In summary, it seems that group II with prolonged FTSST separated from the group of patients with correct HGS presents numerous LVDd parameters, as well as impaired systolic function of the left and right ventricles, additionally to metabolic disturbances, and perhaps should be classified as probable sarcopenia. This is due to the fact that muscle strength measured by FTSST is more sensitive than HGS in the prediction of LVDd, as well as systolic function of the left and right ventricles.

The main limitation of our study is the small number of only male participants. However, it is a pilot study, the results of which allowed us to plan similar research involving larger numbers of patients of both sexes. The reason for recruiting only men was to create the samples which would be homogeneous in terms of body composition and hormonal profile. Future study should also include the assessment of albuminuria, the only recommended biomarker of cardiovascular risk in CKD.

## 5. Conclusions

In the population of G4–G5 CKD patients not treated with dialysis, HGS and FTSST are not equivalent and should not be used interchangeably as is recommended in the general elderly population. Decreased muscle strength is associated with LVDd in G4–G5 CKD patients not treated with dialysis and FTSST is more sensitive than HGS in the prediction of LVDd in this group of patients. Low muscle strength is also associated with systolic function of the left and right ventricles in G4–G5 CKD patients not treated with dialysis. These findings should be confirmed in a larger study.

## Figures and Tables

**Table 1:** Clinical and anthropometric characteristics of the studied samples.

	HGS < 27 kg (n = 23)	HGS ≥ 27 kg (n = 23)	*p*-Value **
	Mean ± SD *	Mean ± SD *	
Age [years]	65.17 ± 11.35	63.48 ± 9.89	0.524
HGS [kg]	22.00 ± 3.82	36.58 ± 6.87	**<0.001**
FTSST [s]	19.95 ± 5.29	14.73 ± 4.72	**0.001**
Serum creatinine [mg/dL]	3.3 ± 0.77	3.17 ± 0.63	0.620
eGFR [mL/min/1.73 m^2^]	20.98 ± 4.68	22.04 ± 4.88	0.481
Serum glucose [mg/dL]	105.48 ± 30.75	111.87 ± 45.82	0.926
Insulin [µU/mL]	10.60 (4.90;19.40)	9.70 (7.00;22.60)	0.740
HOMA-IR	2.44 (1.49;4.36)	2.34 (1.64;6.53)	0.896
Haemoglobin [g/dL]	11.97 ± 2.18	12.27 ± 1.77	0.675
Serum albumin [g/dL]	4.16 ± 0.36	4.35 ± 0.54	0.055
Total cholesterol [mg/dL]	151.13 ± 34.23	166.30 ± 42.33	0.106
Serum testosterone [ng/mL]	3.42 ± 1.70	3.89 ± 1.55	0.475
Weight [kg]	84.01 ± 14.37	87.87 ± 13.56	0.386
Height [cm]	173.22 ± 6.67	175.26 ± 6.88	0.385
BMI [kg/m^2^]	27.97 ± 4.38	28.66 ± 4.51	0.659
LTM [%]	47.11 ± 8.57	49.38 ± 11.93	0.691
LTI [kg/m^2^]	12.94 ± 2.34	13.83 ± 2.41	0.531
Fat [%]	36.47 ± 6.19	35.43 ± 8.98	0.944
FTI [kg/m^2^]	13.86 ± 3.44	14.12 ± 5.18	0.840
ASM [kg]	22.14 ± 4.31	23.31 ± 3.33	0.315
OH [L]	1.98 ± 1.98	1.39 ± 1.77	0.235

HGS, hand grip strength; FTSST, five-times sit-to-stand test; eGFR, estimated glomerular filtration rate; HOMA-IR, homeostasis model assessment of insulin resistance; BMI, body mass index; LTM, lean tissue mass; LTI, lean tissue index; FTI, fat tissue index; ASM, appendicular skeletal muscle mass; OH, overhydration; *p*-values < 0.05 are marked in bold. (*) for insulin and HOMA-IR median (IQR). (**) exact Mann–Whitney test.

**Table 2:** The characteristics of the studied samples according to the Mini Sarcopenia Risk Assessment including 7-item questionnaire.

		HGS < 27 kg (n = 21)	HGS ≥ 27 kg (n = 8)	*p*-Value *
1st question	Age ≥ 70 years	52.2%	43.5%	0.555
2nd question	≥1 hospitalisation in the last year	56.5%	26.1%	**0.036**
3rd question	Not able to walk more than 1000 m	56.5%	13.0%	**0.002**
4th question	Does not consume regularly 3 meals per day	13.0%	8.7%	1.000
5th question	Does not eat milk or dairy products every day	73.9%	78.3%	0.730
6th question	Does not eat poultry, meat, fish, eggs, legumes, ragout, or ham every day	56.5%	65.2%	0.546
7th question	Lost more than 2 kg in the last year	47.8%	34.8%	0.369

HGS, hand grip strength; *p*-values < 0.05 are marked in bold. (*) chi-square test (Fisher test).

**Table 3:** The characteristics of the studied population according to echocardiographic parameters.

	HGS < 27 kg (n = 20)	HGS ≥ 27 kg (n = 23)	*p*-Value *
	Mean ± SD	Mean ± SD	
LA [mm]	41.85 ± 6.54	42.87 ± 5.40	0.509
LA [cm^2^]	22.20 ± 5.63	24.35 ± 5.99	0.294
LAVI [mL/m^2^]	23.16 ± 8.96	27.84 ± 10.24	0.149
LAVI ≥ 29 mL/m^2^ [%]	20.0	34.8	0.281
IVS [mm]	11.60 ± 1.50	11.57 ± 1.59	0.928
PWD [mm]	11.15 ± 1.23	10.74 ± 1.51	0.250
LVEF [%]	56.30 ± 5.44	56.74 ± 9.26	0.449
LVEF < 50 [%]	10.0	17.4	0.669
E [cm/s]	70.95 ± 20.65	73.77 ± 12.87	0.252
E/A	0.91 ± 0.42	0.99 ± 0.41	0.413
Septal e’ [cm/s]	6.75 ± 1.97	7.68 ± 1.96	0.110
Septal e’ < 7 cm/s [%]	50.0	31.8	0.231
Lateral e’ [cm/s]	8.50 ± 3.09	9.23 ± 3.22	0.494
Lateral e’ < 10 cm/s [%]	65.0	63.6	0.927
Average e’ [cm/s]	7.62 ± 2.16	8.86 ± 3.20	0.160
Average septal–lateral E/e’ ratio	9.81 ± 3.29	9.30 ± 4.08	0.358
Average septal–lateral E/e’ ratio ≥ 9 [%]	55.0	40.9	0.361
LVMI [g/m^2^]	121.42 ± 38.74	128.43 ± 37.35	0.763
LVMI > 115 g/m^2^ [%]	60.0	60.9	0.954
RWT	0.45 ± 0.06	0.41 ± 0.06	0.051
RWT > 0.42 [%]	65.0	39.1	0.091
TAPSE [mm]	22.40 ± 4.55	24.09 ± 4.45	0.235
TAPSE ≤ 18 mm [%]	15.0	8.7	0.650
S’ wave [cm/s]	13.85 ± 3.51	14.30 ± 2.88	0.889

LA [mm], left atrial anteroposterior dimension; LA [cm^2^], left atrial surface area measured in the parasternal long-axis view; LAVI, left atrial volume index; IVS, interventricular septum; PWD, posterior wall diameter; LVEF, left ventricular ejection fraction; E wave, the maximal velocity of early diastolic transmitral inflow; E/A, the ratio of the maximal velocity of early diastolic transmitral inflow and the maximal velocity through mitral valve in the later atrial contraction; septal e’, early diastolic velocity at the septal mitral annulus; lateral e’, early diastolic velocity at the lateral mitral annulus; average e’ = (septal e’ + lateral e’)/2; LVMI, left ventricular mass index; RWT, relative wall thickness; TAPSE, tricuspid annular plane systolic excursion; S’ wave, pulsed wave–Doppler tissue imaging (PW–DTI)-derived tricuspid lateral annular peak systolic velocity; (*) exact Mann–Whitney test.

**Table 4:** Subgroup analysis according to echocardiographic parameters.

	HGS < 27 kg FTSST > 15 s (I Group)		HGS ≥ 27 kg FTSST > 15 s (II Group)		HGS ≥ 27 kg FTSST ≤ 15 s (III Group)		*p*-Value *
	n	Mean ± SD	n	Mean ± SD	n	Mean ± SD	
LA [mm]	18	42.39 ± 6.36	8	45.38 ± 5.07	15	41.53 ± 5.24	0.303
LA [cm^2^]	18	22.83 ± 5.57	8	27.13 ± 5.96	15	22.87 ± 5.64	0.146
LAVI [mL/m^2^]	18	23.97 ± 9.07	8	31.23 ± 8.96	15	26.03 ± 10.70	0.173
LAVI ≥ 29 mL/m^2^ [%]	18	22.2	8	50.0	15	26.7	0.361
IVS [mm]	18	11.61 ± 1.58	8	12.25 ± 1.39	15	11.2 ± 1.61	0.265
PWD [mm]	18	11.17 ± 1.29	8	11.00 ± 1.77	15	10.60 ± 1.40	0.473
LVEF [%]	18	56.28 ± 5.75	8	52.00 ± 7.45 ^a^	15	59.27 ± 9.35 ^a^	**0.021**
LVEF < 50 [%]	18	11.1	8	37.5	15	6.7	0.171
E wave [cm/s]	18	71.17 ± 21.36	7	81.71 ± 12.37	15	70.07 ± 11.69	0.102
E/A	18	0.91 ± 0.43	7	1.06 ± 0.50	15	0.96 ± 0.37	0.764
Septal e’ [cm/s]	18	6.50 ± 1.79 ^b^	7	6.43 ± 2.07	15	8.27 ± 1.67 ^b^	**0.016**
Septal e’ < 7 cm/s [%]	18	55.6	7	57.1	15	20.0	0.093
Lateral e’ [cm/s]	18	8.50 ± 3.26	7	8.00 ± 3.06	15	9.80 ± 3.23	0.407
Lateral e’ < 10 cm/s [%]	18	61.1	7	71.4	15	60.0	1.000
Average e’ [cm/s]	18	7.49 ± 2.20	7	7.21 ± 2.41	15	9.63 ± 3.30	0.057
Average septal–lateral E/e’ ratio	18	10.03 ± 3.40	7	12.60 ± 5.32 ^c^	15	7.75 ± 2.20 ^c^	**0.030**
Average septal–lateral E/e’ ratio ≥ 9 [%]	18	61.1	7	57.1	15	33.3	0.284
LVMI [g/m^2^]	18	126.23 ± 37.83	8	141.78 ± 31.63	15	121.31 ± 39.18	0.340
LVMI > 115 g/m^2^ [%]	18	66.7	8	87.5	15	46.7	0.143
RWT	18	0.45 ± 0.06	8	0.41 ± 0.07	15	0.42 ± 0.06	0.251
RWT > 0.42 [%]	18	61.1	8	50.0	15	33.3	0.311
TAPSE [mm]	18	22.78 ± 4.32	8	20.63 ± 2.77 ^d^	15	25.93 ± 4.10 ^d^	**0.017**
TAPSE ≤ 18 mm [%]	18	11.1	8	25.0	15	0.0	0.170
S’ wave [cm/s]	18	14.28 ± 3.37	8	12.63 ± 2.13	15	15.20 ± 2.88	0.123

LA [mm], left atrial anteroposterior dimension; LA [cm^2^], left atrial surface area measured in the parasternal long-axis view; LAVI, left atrial volume index; IVS, interventricular septum; PWD, posterior wall diameter; LVEF, left ventricular ejection fraction; E wave, the maximal velocity of early diastolic transmitral inflow; E/A, the ratio of the maximal velocity of early diastolic transmitral inflow and the maximal velocity through mitral valve in the later atrial contraction; septal e’, early diastolic velocity at the septal mitral annulus; lateral e’, early diastolic velocity at the lateral mitral annulus; average e’ = (septal e’+ lateral e’)/2; LVMI, left ventricular mass index; RWT, relative wall thickness; TAPSE, tricuspid annular plane systolic excursion; S’ wave, pulsed wave–Doppler tissue imaging (PW–DTI)-derived tricuspid lateral annular peak systolic velocity; *p*-values < 0.05 are marked in bold. (*) exact Kruskal–Wallis test. (^a^) *p* = 0.022 in pairwise comparisons adjusted by the Bonferroni correction for multiple tests. (^b^) *p* = 0.024 in pairwise comparisons adjusted by the Bonferroni correction for multiple tests. (^c^) *p* = 0.047 in pairwise comparisons adjusted by the Bonferroni correction for multiple tests. (^d^) *p* = 0.023 in pairwise comparisons adjusted by the Bonferroni correction for multiple tests.

**Table 5:** Subgroup analysis according to clinical and biochemical variables.

	HGS < 27 kg FTSST > 15 s (I Group) (n = 21)	HGS ≥ 27 kg FTSST > 15 s (II Group) (n = 8)	HGS ≥ 27 kg FTSST ≤ 15 s (III Group) (n = 15)	*p*-Value **
	Mean ± SD *	Mean ± SD *	Mean ± SD *	
Age [years]	66.05 ± 10.49	67.5 ± 6.21	61.33 ± 10.97	0.306
HGS [kg]	21.96 ± 3.84 ^a,b^	32.41 ± 4.35 ^a^	38.80 ± 7.03 ^b^	**<0.001**
FTSST [s]	20.71 ± 4.87 ^c^	20.31 ± 3.00 ^d^	11.75 ± 1.81 ^c,d^	**<0.001**
Serum creatinine [mg/dL]	3.26 ± 0.77	3.03 ± 0.63	3.24 ± 0.64	0.757
eGFR [mL/min/1.73 m^2^]	21.17 ± 4.77	22.38 ± 4.93	21.87 ± 5.01	0.819
Serum glucose [mg/dL]	109.57 ± 28.93	138.88 ± 62.07	97.47 ± 27.10	0.075
Insulin [µU/mL]	8.50 (4.75;17.95)	13.00 (9.40; 26.65)	9.20 (5.60;14.20)	0.214
HOMA-IR	2.17 (1.22;4.46)	4.43 (2.45;11.57)	2.04 (1.23;3.26)	0.075
Haemoglobin [g/dL]	12.06 ± 2.23	12.33 ± 1.53	12.25 ± 1.93	0.960
Serum albumin [g/dL]	4.14 ± 0.37	4.43 ± 0.62	4.31 ± 0.50	0.157
Total cholesterol [mg/dL]	151.57 ± 35.54	165.00 ± 33.57	167.00 ± 47.45	0.292
Serum testosterone [ng/mL]	3.34 ± 1.50	4.15 ± 1.79	3.75 ± 1.45	0.688
Weight [kg]	84.15 ± 14.54	94.18 ± 9.14	84.50 ± 14.57	0.090
Height [cm]	173.62 ± 6.84	178.00 ± 6.19	173.80 ± 6.97	0.475
BMI [kg/m^2^]	27.89 ± 4.40	29.81 ± 3.59	28.04 ± 4.93	0.280
LTM [%]	47.39 ± 8.65	44.78 ± 5.87	51.84 ± 13.71	0.381
LTI [kg/m^2^]	13.07 ± 2.30	13.35 ± 2.37	14.09 ± 2.46	0.628
Fat [%]	36.22 ± 6.24	38.78 ± 4.12	33.65 ± 10.42	0.303
FTI [kg/m^2^]	13.87 ± 3.59	15.70 ± 2.49	13.28 ± 6.07	0.342
ASM [kg]	22.19 ± 4.22	24.38 ± 2.89	22.74 ± 3.51	0.226
OH [L]	2.00 ± 2.07	1.38 ± 1.64	1.39 ± 1.89	0.508

HGS, hand grip strength; FTSST, five-times sit-to-stand test; eGFR, estimated glomerular filtration rate; HOMA-IR, homeostasis model assessment of insulin resistance; BMI, body mass index; LTM, lean tissue mass; LTI, lean tissue index; FTI, fat tissue index; ASM, appendicular skeletal muscle mass; OH, overhydration; *p*-values < 0.05 are marked in bold. (*) for insulin and HOMA-IR median (IQR). (**) exact Kruskal–Wallis test. (^a^) *p* = 0.003 in pairwise comparisons adjusted by the Bonferroni correction for multiple tests. (^b^) *p* < 0.001 in pairwise comparisons adjusted by the Bonferroni correction for multiple tests. (^c^) *p* < 0.001 in pairwise comparisons adjusted by the Bonferroni correction for multiple tests (^d^) *p* < 0.001 in pairwise comparisons adjusted by the Bonferroni correction for multiple tests.

**Table 6:** Subgroup analysis according to the Mini Sarcopenia Risk Assessment 7-item questionnaire.

		HGS < 27 kg FTSST > 15 s (I Group) (n = 21)	HGS ≥ 27 kg FTSST > 15 s (II Group) (n = 8)	HGS ≥ 27 kg FTSST ≤ 15 s (III Group) (n = 15)	*p*-Value *
1st question	Age ≥ 70 years	57.1%	62.5%	33.3%	0.331
2nd question	≥1 hospitalisation in the last year	52.4%	12.5%	33.3%	0.137
3rd question	Not able to walk more than 1000 m	57.1% ^a,b^	12.5% ^a^	13.3% ^b^	**0.008**
4th question	Does not consume regularly 3 meals per day	9.5%	12.5%	6.7%	1.000
5th question	Does not eat milk or dairy products every day	71.4%	87.5%	73.3%	0.808
6th question	Does not eat poultry, meat, fish, eggs, legumes, ragout, or ham every day	52.4%	50.0%	73.3%	0.435
7th question	Lost more than 2 kg in the last year	47.6%	50.0%	26.7%	0.435

HGS, hand grip strength; FTSST, five-times sit-to-stand test; *p*-values < 0.05 are marked in bold. (*) Fisher test. (^a^) *p* = 0.044 in pairwise comparisons adjusted by the Bonferroni correction for multiple tests. (^b^) *p* = 0.014 in pairwise comparisons adjusted by the Bonferroni correction for multiple tests.

## Data Availability

Original data can be requested from the corresponding author upon reasonable request.

## Abbreviations

AGEs	advanced glycation end-products
ASM	appendicular skeletal muscle mass
Average e’	septal e’ + lateral e’)/2
BCG	bromocresol green
BCM	body composition monitor
BIS	bioimpedance spectroscopy
BMI	body mass index
CHOD-PAP	enzymatic colorimetric
CKD	chronic kidney disease
CKD-EPI	Chronic Kidney Disease Epidemiology Collaboration
CST	chair stand test
eGFR	estimated glomerular filtration rate
E/A	the ratio of the maximal velocity of early diastolic transmitral inflow and the maximal velocity through mitral valve in the later atrial contraction
ECLIA	electrochemiluminescence immunoassay
E/e’	average septal-lateral E/e’ ratio
E wave	the maximal velocity of early diastolic transmitral inflow
EWGSOP2	European Working Group on Sarcopenia in Older People
Fat	fat mass
FGF23	fibroblast growth factor 23
FTI	fat tissue index
FTSST	five-times sit-to-stand test
GH	growth hormone
GH/IGF-1	insulin-like growth factor-1
HF	heart failure
HFpEF	heart failure with preserved ejection fraction
HGS	hand grip strength
HOMA-IR	homeostasis model assessment of insulin resistance
IVS	interventricular septum
LA [mm]	left atrial anteroposterior dimension
LA [cm^2^]	left atrial surface area measured in the parasternal long-axis view
LAVI	left atrial volume index
lateral e’	early diastolic velocity at the lateral mitral annulus
LVDd	left ventricular diastolic dysfunction
LVEF	left ventricular ejection fraction
LTI	lean tissue index
LTM	lean tissue mass
LVMI	left ventricular mass index
NT-proBNP	N-terminal pro-B-type natriuretic peptide
OH	overhydration
PL-MSRA-7	Polish validated version of the Mini Sarcopenia Risk Assessment Questionnaire
PWD	posterior wall diameter
RWT	relative wall thickness
septal e’	early diastolic velocity at the septal mitral annulus
SLS	sodium lauryl sulfate
S’ wave	pulsed wave–Doppler tissue imaging (PW–DTI)-derived tricuspid lateral annular peak systolic velocity
TAPSE	tricuspid annular plane systolic excursion
